# Profound Reprogramming towards Stemness in Pancreatic Cancer Cells as Adaptation to AKT Inhibition

**DOI:** 10.3390/cancers12082181

**Published:** 2020-08-05

**Authors:** Hugo Arasanz, Carlos Hernández, Ana Bocanegra, Luisa Chocarro, Miren Zuazo, Maria Gato, Karina Ausin, Enrique Santamaría, Joaquín Fernández-Irigoyen, Gonzalo Fernandez, Eva Santamaria, Carlos Rodríguez, Idoia Blanco-Luquin, Ruth Vera, David Escors, Grazyna Kochan

**Affiliations:** 1 Oncoimmunology, Navarrabiomed-UPNA, Navarra Institute for Health Research (IdiSNA), Irunlarrea 3, 31008 Pamplona, Spain; hugo.arasanz.esteban@navarra.es (H.A.); chernans77@gmail.com (C.H.); ai.bocanegra.gondan@navarra.es (A.B.); luisa.chocarro.deerauso@navarra.es (L.C.); miren.zuazo.ibarra@navarra.es (M.Z.); mgato@unav.es (M.G.); idoia.blanco.luquin@navarra.es (I.B.-L.); 2 Medical Oncology Unit, Complejo Hospitalario de Navarra (CHN), IdISNA, Irunlarrea 3, 31008 Pamplona, Spain; gonzalo.fernandez.hinojal@navarra.es (G.F.); ruth.vera.garcia@navarra.es (R.V.); 3 Proteored-ISCIII, Proteomics Platform, Navarrabiomed, Complejo Hospitalario de Navarra (CHN), Universidad Pública de Navarra (UPNA), IdISNA, Irunlarrea 3, 31008 Pamplona, Spain; karina.ausin.perez@navarra.es (K.A.); enrique.santamaria.martinez@navarra.es (E.S.); Joaquin.fernandez.irigoyen@navarra.es (J.F.-I.); 4 Centro de Investigación Biomédica en Red de Enfermedades Hepáticas y Digestivas (CIBERehd), CIMA, Universidad de Navarra, IdISNA, Irunlarrea 3, 31008 Pamplona, Spain; evasmaria@unav.es (E.S.); crodriguez@unav.es (C.R.)

**Keywords:** Pancreatic cancer, cancer stem cell, AKT

## Abstract

Cancer cells acquire resistance to cytotoxic therapies targeting major survival pathways by adapting their metabolism. The AKT pathway is a major regulator of human pancreatic adenocarcinoma progression and a key pharmacological target. The mechanisms of adaptation to long-term silencing of AKT isoforms of human and mouse pancreatic adenocarcinoma cancer cells were studied. Following silencing, cancer cells remained quiescent for long periods of time, after which they recovered proliferative capacities. Adaptation caused profound proteomic changes largely affecting mitochondrial biogenesis, energy metabolism and acquisition of a number of distinct cancer stem cell (CSC) characteristics depending on the AKT isoform that was silenced. The adaptation to AKT1 silencing drove most de-differentiation and acquisition of stemness through C-MYC down-modulation and NANOG upregulation, which were required for survival of adapted CSCs. The changes associated to adaptation sensitized cancer cells to inhibitors targeting regulators of oxidative respiration and mitochondrial biogenesis. In vivo pharmacological co-inhibition of AKT and mitochondrial metabolism effectively controlled pancreatic adenocarcinoma growth in pre-clinical models.

## 1. Introduction

Phosphatidyl inositol 3-phosphotase kinase/AKT/molecular target of rapamycin (PI3K/AKT/mTOR) signaling axis constitutes a central pathway involved in progression of pancreatic adenocarcinoma. The main regulators of this pathway are the AKT kinases, which contribute to carcinogenesis, proliferation, migration, angiogenesis and cell survival. However, AKT-mTOR-targeted therapies are clinically ineffective as tumor cells adapt to overcome inhibition of this key signaling axis [1,2,3].

Classically, escape of tumor cells from cytotoxic therapies has been associated to selection of variants with resistance mutations present within a heterogeneous population of tumor cells. However, recent evidence indicates that treatment-resistant tumors arise from a population of cancer stem cells (CSC) derived from within a collection of metabolically plastic cancer cells in the tumor [4]. Indeed, whether treatment-resistant cells come from a pre-existing small population of CSCs or they are selected by metabolic adaptation through a process of de-differentiation is still a matter of debate. Moreover, therapy-resistant cancer cells can also be selected by other non-genetic mechanisms that include the influence of specific tumor microenvironments through epigenetic regulation [5], distinct mitochondrial content [6,7] and metabolic reprogramming such as the switch from mitochondrial oxidative phosphorylation (OXPHOS) to oxygen-independent glycolysis (Warburg Effect) [8]. Therefore, it is yet unclear whether inhibition of pro-survival pathways can force cancer cells to directly alter their metabolism through a process of adaptation leading to de-differentiation towards CSCs.

AKT1, AKT2 and AKT3 are three kinase variants with striking similar sequence conservation. Recent studies indicate that these isoforms do not play redundant roles, but possess specific functions. However, in many instances, it is difficult to ascribe the contribution of each isoform to the biology of the cancer cell. While AKT1 is pro-tumorigenic in lung cancer and ErbB2 positive breast cancer [9,10], its silencing is pro-tumorigenic in prostate and other types of breast cancer [11,12]. In murine breast cancer models, AKT2 abrogation suppresses cell migration, and its expression stimulates motility and invasion in prostate cancer cells in vitro and in breast and ovarian cancer in vivo. In contrast, AKT1 and AKT3 have differing effects in these experimental models [12,13]. In murine lung cancer models, both AKT2 and AKT3 had anti-proliferative and pro-apoptotic properties [9,10]. AKT3 inhibition caused apoptosis and inhibited tumor progression and growth in vivo in melanoma and in breast cancer [14].

In pancreatic adenocarcinoma, the consensus is that AKT activities are carcinogenic and pro-tumorigenic. However, there are conflicting results on the exact role of each specific AKT isoform. Here, we studied the mechanism of adaptation of human pancreatic cancer cells to sustained inhibition of AKT isoforms, whether this can drive cancer cell de-differentiation towards CSCs and how this process occurs.

## 2. Results

### 2.1. Adaptation of Cancer Cells to Silencing of AKT Isoforms Triggers Major Changes in Their Proteome

**Figure 1:**
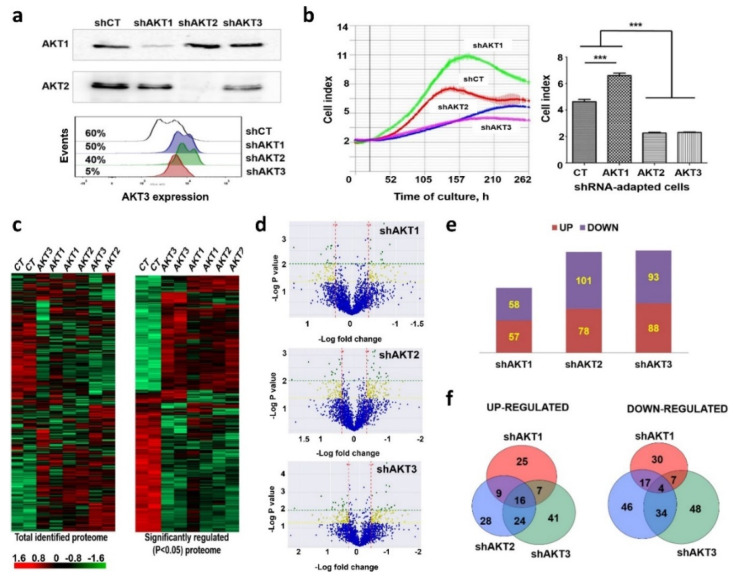
Adaptation of human pancreatic adenocarcinoma cells to specific silencing of AKT isoforms. (**a**) Immunoblots on top, detection of the indicated AKT kinases in cell lines constitutively expressing the shRNA named on top. Original uncropped Western blots and densitometry readings are shown in Figures S1 and S2. Below, flow cytometry histogram of AKT3 expression in the cell lines expressing the indicated shRNAs shown on the right. Percentages of AKT3-expressing cells are shown. The results shown correspond to analyses after more than 20 cell passages after adaptation. (**b**) Left, RTCA graph depicting the growth of the cell lines expressing the indicated shRNAs after adaptation. Right, column graph plotting Cell Index from RTCA data, with means and standard deviations as error bars (four replicates). Relevant statistical comparisons are shown by ANOVA and Tukey’s pairwise tests. (**c**) Left, cluster analysis of the complete identified proteome from cells adapted to silencing of the indicated AKT isoforms, shown as a heat map. Right, same as left but with significantly regulated (*p* < 0.05) proteins as identified by ANOVA. Red, upregulated genes; Green, downregulated genes; Black, unchanged expression. (**d**) Volcano-plots representing fold-change differences for identified proteins using pair-wise comparisons between shCT-control cells and cell lines adapted to silencing of the indicated AKT isoforms as shown on top of each plot. Blue, proteins without significant changes; Yellow, differentially expressed proteins, *p* < 0.05; Green, differentially expressed proteins, *p* < 0.01. (**e**) bar graph of the number of significantly upregulated (red) and downregulated (blue) proteins identified in the volcano plots. (**f**) Venn diagrams of differentially upregulated (left) or downregulated (right) proteins in the indicated cell lines with each silenced AKT isoform. Overlaps are shown, together with the number of overlapping identified proteins indicated within the circles. shCT, control shRNA; ***, highly significant (*p* < 0.001) differences.

The PI3K-AKT signaling axis is one of the major pathways associated to pancreatic cancer cell progression. However, small molecule inhibitors have demonstrated poor clinical performance. Hence, we decided to investigate the mechanisms by which cancer cells become resistant to its prolonged inhibition by interfering with the expression of each of the AKT isoforms, hoping that this information may also identify the most critical isoform for adaptation. Therefore, cell lines were generated from the human pancreatic ductal adenocarcinoma cell line AsPC-1 in which each AKT kinase isoform was individually silenced. AsPC-1 cells harbor mutations identified in the majority of human adenocarcinomas (K-RAS G35A, p53/p16 inactivation). Lentivectors encoding AKT-targeted shRNAs or shCT (an irrelevant shRNA control) together with puromycin resistance were used to transduce cells and recover puromycin resistant clones. The growth of puromycin-resistant cells was stalled for two months following silencing, after which the cells recovered proliferative capacities. Re-expression of AKT kinases was discarded as an escape mechanism throughout the duration of the study by evaluating silencing of AKT isoforms by Western blot at late passages (Figure 1a). Growth kinetics of adapted cell lines were studied by real-time cell monitoring (RTCA). Cell lines adapted to AKT2 and AKT3 silencing still proliferated significantly less than control cells. In contrast, cells adapted to AKT1 silencing recovered higher proliferative capacities than control AKT-non-silenced cells (Figure 1b).

To identify the changes that had occurred as a result of adaptation, the proteomes of each cell line were analyzed by quantitative differential proteomics. In total, 3930 proteins were identified with a false discovery rate (FDR) lower than 1%. Cluster analysis of the identified proteomes uncovered major changes that separated cells adapted to silencing of AKT isoforms from control cells expressing an irrelevant shRNA (Figure 1c). Significantly regulated proteins were identified for each cell line (Figure 1c). Pair-wise comparisons showed significant quantitative differences between the proteomes (Figure 1d). Overall, 115 proteins were differentially regulated in cells adapted to AKT1 silencing compared to control cells (*p* < 0.05), while 179 and 181 were differentially expressed in cells with silenced AKT2 or AKT3, respectively (Figure 1e). Only a minority of down- or upregulated proteins were shared by cells adapted to silencing of any isoform (Figure 1f). These results indicated that adapted cells had undergone specific proteomic profile changes.

### 2.2. Adaptation of Cancer Cells to Silencing of AKT Isoforms Causes Profound Mitochondrial Alterations

**Figure 2:**
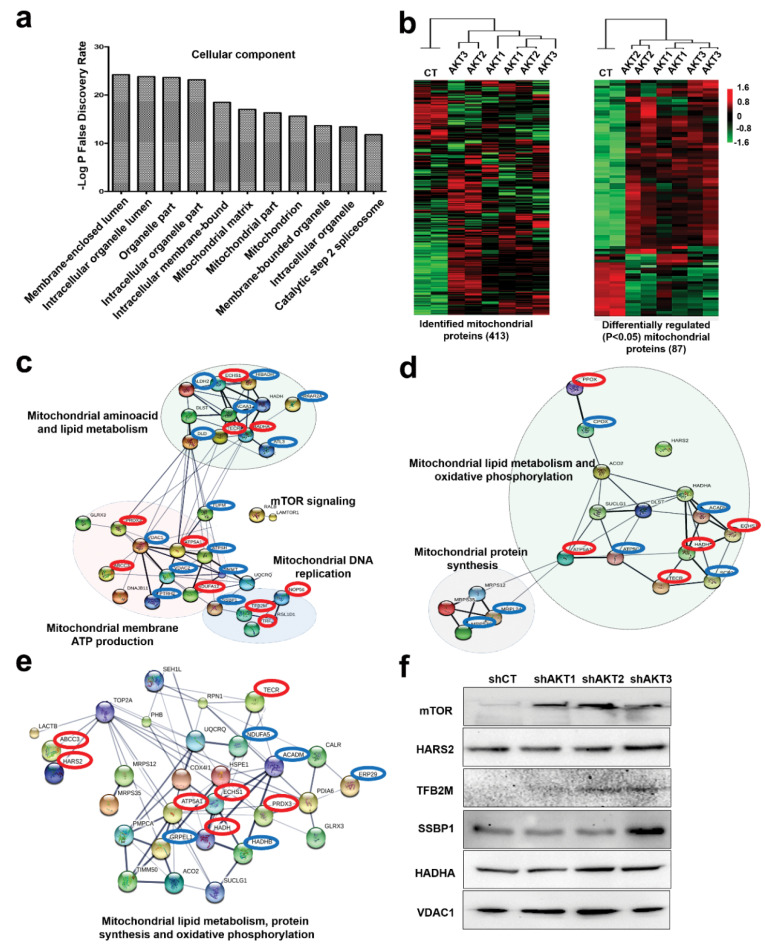
The mitochondrial proteome is significantly altered in cells adapted to AKT silencing. (**a**) Bar graph with the top ten main cellular components in adapted cells to AKT silencing identified using STRING. (**b**) Left, cluster analysis of the identified mitochondrial proteome using two biological replicates per cell line (shown on top), represented by a heatmap. Right, the same as left but with significantly (*P* < 0.05) regulated mitochondrial proteins between the cell lines (*P* < 0.05) identified by ANOVA. (**c**) Functional interactomes with significantly upregulated proteins (*P* < 0.05) in cells adapted to AKT1 silencing compared to control cells, and to AKT2 silencing (**d**) and AKT3 silencing (**e**). In red, common upregulated proteins present in the three interactomes. In blue, upregulated proteins specific for the adaptation to the indicated AKT kinases. Thin and thick lines shown interactions with medium (0.7) or high (0.9) confidence by STRING. Regulated cellular processes by the functional interactome subgroups are highlighted and indicated. (**f**) Expression of the indicated proteins by Western blot in control cells and in cells adapted to silencing of the indicated AKT isoforms. Original uncropped Western blots and densitometry readings are shown in Figures S3–S8.

Cellular component analyses with differentially regulated proteins for each adapted cell line showed that the most affected organelle was the mitochondrion (Figure 2a). This was confirmed by cluster analysis of the mitochondrion proteome between adapted cells and control cells (Figure 2b). To identify the molecular pathways altered after adaptation compared to control cells, significantly regulated mitochondrial proteins were identified (Figure 2b). Proteins were organized into functional interactomes using STRING and Ingenuity tools, which gave equivalent results. Adaptation to AKT1 silencing increased mTOR regulators and three mitochondrial interactomes associated to ATP production, amino acid metabolism, lipid metabolism and mitochondrial DNA replication (Figure 2c). Adaptation to AKT2 and AKT3 silencing activated different interactomes from AKT1-silenced cells, but these were also associated to the regulation of oxidative phosphorylation and mitochondrial protein synthesis (Figure 2d,e). Overall, adapted cells had potentiated mitochondrial processes. Increased expression of regulators of mitochondrial DNA replication and protein synthesis was suggestive of mitochondrial biogenesis.

We tested the regulation of the mTOR pathway and selected protein targets involved in mitochondrial biogenesis (Figure 2f). Samples were loaded in protein gels based on equal number of cells followed by Ponceau S staining, as standard housekeeping proteins frequently used as loading controls were also differentially regulated in our proteomic data. We confirmed by Western blot that mTOR expression was increased in all adapted cells (Figure 2f), as mTOR activation is a known escape mechanism from AKT inhibition [15] and a major coordinator of mitochondrial activities, protein synthesis and proliferation [16]. Overall, differential expression of the mitochondrial proteins TFB2M, SSBP1 HADHA and VDAC1 was generally confirmed (Figure 2f).

**Figure 3:**
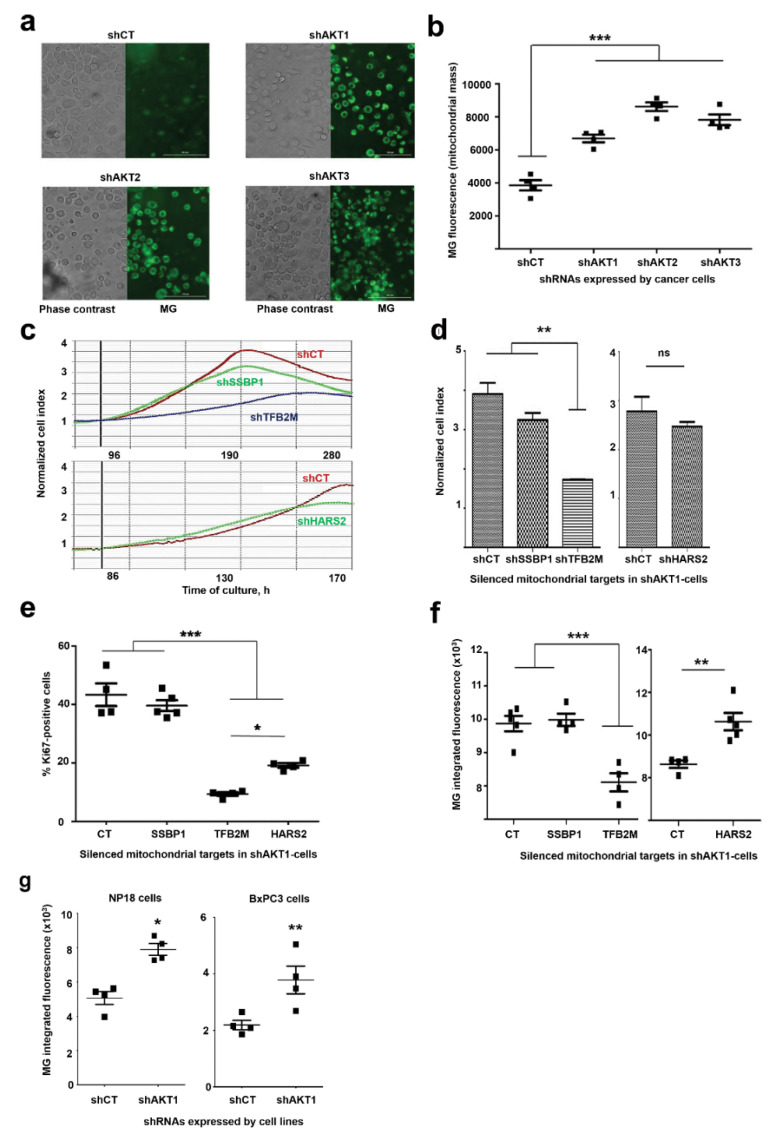
Increased mitochondrial content in cells required for adaptation to AKT silencing. (**a**) Phase contrast and fluorescent microscopy pictures of MitoTracker Green (MG)-stained cancer cell lines adapted to silencing of the indicated AKT isoforms. The control cell line was overexposed to detect background fluorescence signal. Bars within the pictures represent 100 µm. (**b**) Dot plot representing integrated fluorescence intensities, using four biological replicates per cell line. Means and standard deviations are also shown. Relevant statistical comparisons are shown by ANOVA and Tukey’s pairwise tests. (**c**) RTCA graphs representing the growth of cells with silenced AKT1 and additional silencing of the indicated mitochondrial proteins. Data are shown with means from duplicate cultures together with error bars. (**d**) Column graphs plotting the Normalized Cell Index from RTCA data corresponding to the left RTCA graphs. Relevant statistical comparisons are shown in the graph by ANOVA and pair-wise comparisons (Tukey’s test). (**e**) Scatter plot of KI-67 expression by flow cytometry for the indicated cell lines. Relevant statistical comparisons are shown within the graph by ANOVA and Tukey’s pairwise tests. (**f**) Scatter plots of integrated MG fluorescence intensity for the indicated cell lines. Data from five replicates are shown, with means and error bars (standard deviations). (**g**) Scatter plots of integrated MG fluorescence intensity for the indicated cell lines. Data from four replicates are shown, with means and error bars (standard deviations). *, **, ***, indicate significant (*p* < 0.05), very significant (*p* < 0.01) and highly significant (*p* < 0.001) differences.

To confirm that adaptation caused an increase in mitochondrial mass, MitoTracker Green FM (MG) staining was used as it has been previously demonstrated to be an accurate reporter of mitochondrial mass with negligible phototoxic effects (Figure 3a) [4,17]. Integrated MG intensity in living cells showed a significant increase in mitochondrial mass in adapted cells (Figure 3b). To test if adapted cells relied on mitochondria for growth and survival, we silenced the expression of SSBP1, HARS2 and TFB2M, which regulate mitochondrial DNA replication, protein expression and were upregulated by adaptation to AKT silencing. These targets were silenced with validated shRNAs in AKT1-silenced AsPC-1 cells, as this cell line showed the strongest proliferation and most of the upregulated interactomes were related with mitochondrial functions. Silencing of these mitochondrial targets caused another proliferative arrest that lasted 2–3 months. Nevertheless, the cells recovered proliferative capacities comparable to the original AsPC-1-shAKT1 cell line (Figure 3c) with the exception of cells with silenced TFB2M (Figure 3c,d), which had the most significant negative impact over cell growth, with a concomitant decrease of KI67 expression (Figure 3e). Importantly, MG staining confirmed that only TFB2M silencing had significantly reduced mitochondrial content (Figure 3f). Overall, these results corroborated the proteomic data and that adapted cells relied on mitochondria for growth. To discard that our results were limited to AsPC-1 cells, we adapted two additional humal pancreatic cell lines to prolonged AKT1 silencing, NP18 and BxPC3 cells. Significant increase in mitochondrial mass was confirmed also in these two cell lines by MG staining in cells adapted to AKT1 silencing compared to cells expressing the control shRNA (Figure 3g). These results strongly suggest that increase in mitochondrial mass was a mechanism that could be extended to other pancreatic cancer cells.

### 2.3. Adaptation to AKT Silencing Sensitizes Pancreatic Cancer Cells to Mitochondrial Disrupting Agents

**Figure 4:**
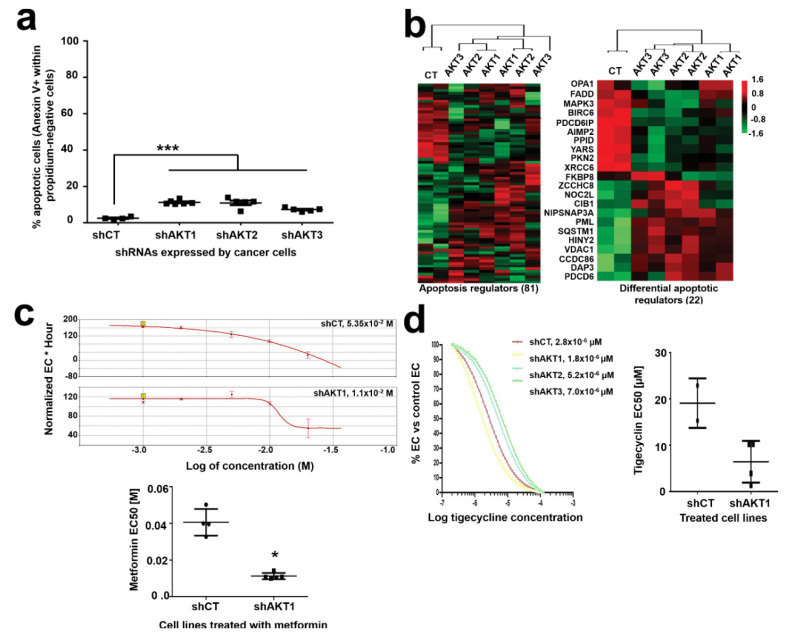
Pancreatic cancer cells with silenced AKT isoforms are sensitive to mitochondria-disrupting agents. (**a**) Dot plot representing the percentage of spontaneous apoptosis in the indicated cell lines. Relevant statistical comparisons are shown by ANOVA and pairwise Tukey’s test. Means and standard deviations are also shown. (**b**) Cluster analysis of proteins involved in regulation of apoptosis (left) and those significantly regulated (right) by silencing of the indicated AKT isoforms. ***, indicate highly significant (*p* < 0.001) differences. (**c**) Top, RTCA EC_50_ (effective concentrations) curves of metformin treatments for the indicated cell lines, four replicates per concentration. The calculated EC_50_s for each graph are shown within the graph. Bottom, dot plot graph of EC_50_ values for metformin treatment calculated from five independent experiments as shown on top, in the indicated cell lines. The statistical comparison was performed with Student’s t test. (**d**) Left, RTCA EC_50_ curves of tigecycline treatments for cell lines with the indicated AKT isoforms silenced, calculated by RTCA. The EC_50_ values are shown. Right, dot plot graph of EC_50_ values for tigecycline calculated from four experiments. *, ***, indicate significant (*p* < 0.05) and highly significant (*p* < 0.001) differences.

It has been previously shown that cells with a higher mitochondrial content exhibit increased apoptosis, which in turn makes them more susceptible to cytotoxic agents [17]. To test whether this was the case for cells adapted to AKT silencing, we quantified the rates of spontaneous apoptosis (Figure 4a). Cells that had adapted to silencing of AKT isoforms showed a slight although significant increase in spontaneous apoptosis compared to control cells. In agreement with this result, cluster analyses of the proteome associated to apoptotic pathways uncovered significant alterations (Figure 4b), with 20 pro- and anti-apoptotic regulators significantly altered. Increased baseline expression of effector caspases 3 and 7 were also found by Western blot in adapted cells. Our results suggest that adapted cells were potentiating mitochondria for survival, which on the other hand could make them more sensitive to apoptosis by mitochondria-disrupting agents. To test whether adapted cells had become sensitized to mitochondria disrupting agents, we evaluated the EC_50_ of metformin in each cell line by RTCA. Metformin is a potent inhibitor of both the respiratory chain complex I and mTOR [18]. The EC_50_ of metformin was reduced in all cells with silenced AKT isoforms compared to control cells as ascertained by RTCA, and it was significant in AKT1-silenced cells compared to shCT control cells (Figure 4c). Tigecycline was then tested as a highly selective inhibitor of the mitochondrial respiratory chain without disrupting the mTOR pathway [19]. Cells adapted to AKT1 silencing exhibited the strongest increase in sensitivity to tigecycline (Figure 4d), although it did not reach statistical significance (Figure 4e). Overall, these results show enhanced capacities of mitochondrial-disrupting agents to counteract the growth of cells adapted to silencing of AKT isoforms.

### 2.4. Adaptation to AKT1 Silencing Causes Acquisition of Cancer Stem Cell Characteristics

**Figure 5:**
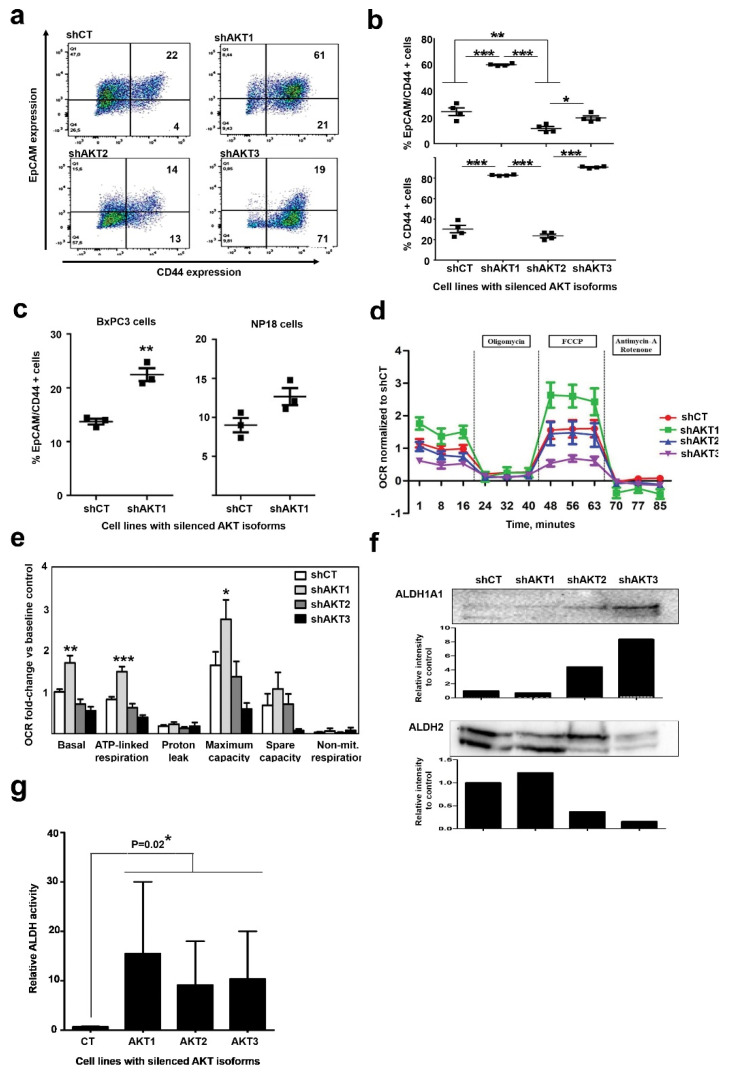
Adapted cells to AKT1 silencing acquire characteristics of cancer stem-like cell. (**a**) Left Flow cytometry density plots of CD44/EpCAM co-expression in the surface of pancreatic cancer cell lines adapted to silencing of the indicated AKT isoform. Percentages of events are shown within each quadrant. (**b**) Scatter plots representing the percentage of cells that co-express CD44/EpCAM (top) or only CD44 (bottom). Data from four biological replicates are shown. Relevant statistical comparisons are shown within the graph, calculated by ANOVA and Tukey’s pairwise tests. (**c**) Scatter plots representing the percentage of cells that co-express CD44/EpCAM for the indicating cell lines. Data from four biological replicates are shown. Relevant statistical comparisons are shown within the graph, calculated by ANOVA and Tukey’s pairwise tests. (**d**) Graph displaying oxygen consumption rates (OCRs) in the indicated cell lines following the addition of oligomycin, FCCP or Antimycin-A/rotenone as shown. Results are shown as means from three technical replicates, together with error bars (SD). (**e**) Bar graph displaying oxygen consumption rate (OCR) for each indicated cell line quantified by Seahorse XF and normalized to baseline OCR from control cells following the addition of the indicated compounds. Data are shown as means and error bars OCR attributed to each mitochondrial function calculated from (C top), and data shown as means and error bars. (**f**) ALDH1A1 and ALDH2 expression by Western blot in the indicated cell lines. Densitometry data on the intensities of both proteins detected by Western blot compared to the controls shCT cell line is shown in the bar graphs below each Western blot. Original uncropped Western blots and densitometry readings are shown in Figures S9 and S10. ALDH2 presents two bands with different electrophoretic mobility due to acetylation. Densitometry was carried out integrating the intensities from both bands. (**g**) Bar graph representing relative ALDH activity quantified by the Aldefluor flow cytometry staining method, with means and standard deviations as error bars. Relevant statistical comparisons are shown by two-way ANOVA, and Tuckey’s pairwise tests. *, **, ***, indicate significant (*p* < 0.05), very significant (*p* < 0.01) and highly significant (*p* < 0.001) differences.

Upregulation of mitochondrial biogenesis and alterations in energy metabolism and mitochondrial respiration by proteomics (Figure 2 and Figure 3), as well as increased sensitivity to metformin and tigecyclin suggested CSC characteristics in adapted cells. To test whether cells had undergone dedifferentiation, we first assessed the expression of CD44 and EpCAM, as these represent the two most accepted CSC-associated markers in pancreatic cancer [4,20] (Figure 5a). Interestingly, cells exhibited differential CD44/EpCAM expression profiles specific for the particular AKT isoform that was silenced (Figure 5b). Pancreatic cancer cells with silenced AKT1 strongly increased the co-expression of these two CSC markers. AKT3 silencing caused increased expression of CD44 but not EpCAM. Cells adapted to AKT2 silencing upregulated neither CD44 nor EpCAM. These results show that AKT1-silenced pancreatic cells resembled CSC much more closely, and that AKT2-silenced cells were the most differentiated. To discard whether acquisition of CSC markers by adaptation to AKT1 silencing was restricted to AsPC-1 cells, we evaluated the upregulation of these markers in NP18 and BxPC3 cells (Figure 5c). Both cell lines co-upregulated CD44/EpCAM, again indicating that adaptation to AKT1 silencing in these cell lines followed a similar mechanism as in AsPC-1 cells.

CSCs are highly dependent on oxidative metabolism [4]. To study the dependence of AsPC-1 cells with silenced AKT isoforms on oxidative metabolism, real-time oxygen consumption was evaluated by Seahorse analyses (Figure 5d). Oxygen consumption by proton efflux, maximal respiration and complete abrogation of mitochondrial respiration were quantified. Only cells with silenced AKT1 exhibited a significant increase in basal and maximal oxygen consumption rates compared to control cells or cells with either AKT2 or AKT3 silenced (Figure 5e).

ALDH activity has been established as a factor responsible for CSC survival, differentiation and resistance to chemo- and radiotherapy. High ALDH1A1 activity has been shown to correlate with CSC phenotype in different cancer types [21]. As our proteomic data show ALDH2 upregulation in cells adapted to AKT1 silencing compared to the other cell lines including the shCT control, we assessed the expression of both ALDH1A1 and ALDH2 by Western blot by loading equal amounts of cells per sample. Interestingly, ALDH1A1 was overexpressed in cells adapted to AKT3 silencing, while high ALDH2 levels were maintained both in cells adapted to AKT1 silencing and control cells (Figure 5f). Nevertheless, ALDH expression levels do not fully correlate with high enzymatic activity as these enzymes can be inhibited by post-translational modifications such as acetylation [22]. Therefore, ALDH activity was quantified in each cell line by flow cytometry using Aldefluor kit (Figure 5g). Interestingly, although there was high variability between experiments, cells adapted to silencing of AKT isoforms showed higher ALDH activities than control cells.

**Figure 6:**
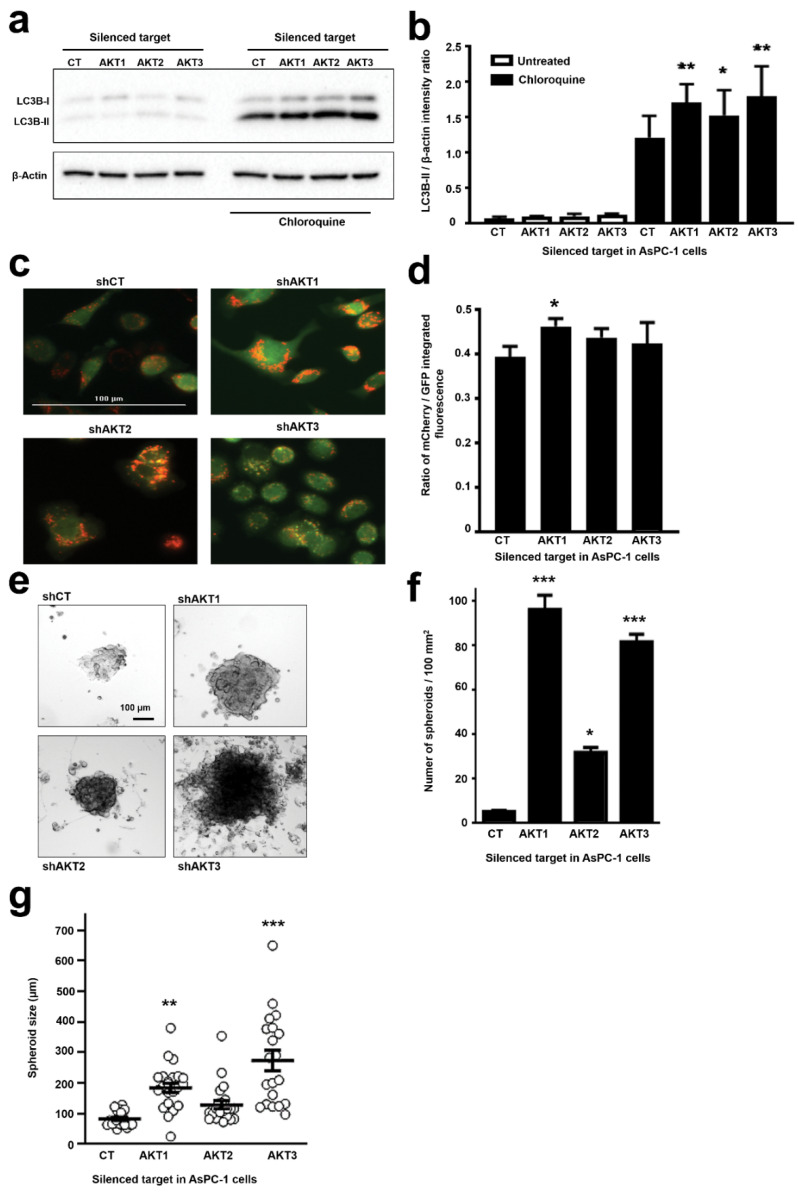
Adaptation of AsPC-1 cells to silencing of AKT isoforms increase autophagy and spheroid growth. (**a**) Western blot with expression of LC3B-I and II forms as indicated in the shown cell lines with or without incubation with chloroquine. Original uncropped Western blots and densitometry readings are shown in the Figures S12 and S13. (**b**) Bar graph with the ratio of intensities for LC3B-II versus β-actin as determined by densitometry of Western blots as in (**a**). Ratios were obtained from triplicates. Statistical comparisons were performed by ANOVA and pairwise Tukey’s tests between AKT-silenced and control groups. (*n* = 3). (**c**) Fluorescence microscopy of living AsPC-1-mCherry-GFP-LC3B cells with silenced AKT isoforms as indicated, or control cells expressing an irrelevant control shRNA (shCT). Representative 20× pictures with merged GFP and mCherry fluorescence. Autophagolysosomes correspond to bright red vesicles. (**d**) The bar graphs represent the ratio of mCherry/GFP mean fluorescence intensities (*n* = 4). Statistical comparisons were performed by ANOVA and pairwise Tukey’s tests. (**e**) Representative phase contrast pictures of spheroids formed from the indicated AsPC-1 cell lines. (**f**) Bar graph shows the number of spheroids per 100 mm^2^ obtained from the indicated cell lines. (**g**) Bar graph shows the spheroid size (*n* = 20) from the indicated cell lines. Statistical comparisons were performed by ANOVA and pairwise Tukey’s test by comparing to control shCT-AsPC-1 cells. *, **, ***, indicate significant (*p* < 0.05), very significant (*p* < 0.01) and highly significant (*p* < 0.001) differences. CT, control shRNA.

CSCs show changes in basal autophagy as it participates in the acquisition and maintenance of stemness and cell survival in the tumor microenvironment. Autophagy also controls CSC potential for migration and invasion, and promotes resistance to anti-cancer therapies [23]. Pancreatic CSCs activate autophagy to maintain stemness [24]. To assess the degree of autophagy in our cell lines, we measured LC3B protein levels by Western blot after incubation with the lysosomal inhibitor chloroquine (Figure 6a). LC3B changes from a cytosolic form (LC3B-I) to an autophagosomal membrane-bound form (LC3B-II) during autophagy. Cells with silenced AKT isoforms accumulated more LC3B-II than control cells, indicating that basal autophagy flux was increased in these cells (Figure 6a,b). However, the loading β-actin control was found to be regulated to some degree in our proteomic data. Therefore, to directly confirm autophagy activation in living cells, we generated AsPC-1 cells expressing a mCherry/GFP/LC3B (mGLC3B) fusion protein tandem. Using this LC3B version, GFP is less stable in the acidic lysosomal compartment than mCherry, and autophagolysosomes can be easily identified as bright red vesicles by fluorescence microscopy in living cells. Hence, AKT isoforms were silenced in AsPC1-mGLC3B cells, and they were visualized (Figure 6c). Increased number of autophagolysosomes was observed by microscopy especially when AKT1 and AKT2 were silenced. To have a quantitative estimation, we took advantage that in this system autophagy levels proportionally correlate with mCherry/GFP fluorescence ratios [25]. Cells were analyzed by fluorescence microscopy (Cytation 5), and, compared to control cells, all cells with silenced AKT isoforms exhibited a higher ratio of mCherry/GFP mean fluorescence intensity, corroborating the increase in autolysosomes (Figure 6d). This increase reached statistical significance for cells with silenced AKT1.

One of the most important characteristics of stemness is the increased capacity of cells to grow as spheroids [26]. Hence, the capacities of the different AsPC-1 cell lines to grow as spheroids were tested. While all cell lines including shCT-AsPC-1 cells formed spheroids (Figure 6e), cells adapted to silencing of AKT isoforms formed more numerous (Figure 6f) and larger spheroids per 100 mm^2^ (Figure 6g). Interestingly, the spheroid-forming capacities were significantly higher for AsPC-1 cells with silenced AKT1 or AKT3.

### 2.5. Adaptation to AKT1 Silencing Uncovers Specific Regulation of C-MYC and NANOG

**Figure 7:**
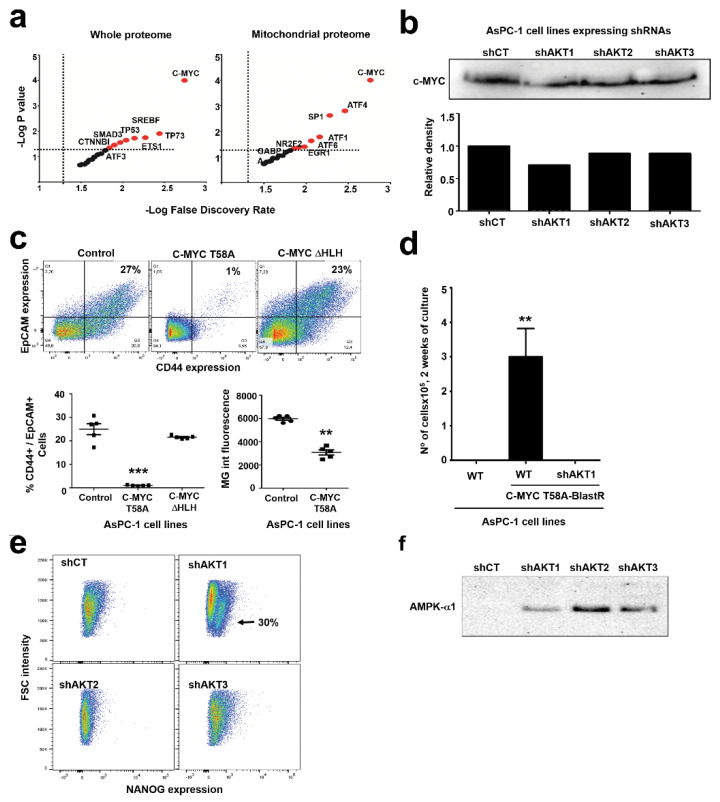
Adaptation to AKT1 silencing uncovers specific regulation of C-MYC, NANOG and AMPK. (**a**) Dot plots of transcription factors (indicated within the graph) associated to potential regulation of the proteomes associated to silencing of AKT isoforms compared to shCT-control cancer cells. The left graph represents data from the whole differential proteomes, while the right graph represents data from the mitochondrial differential proteomes. Horizontal and vertical dotted lines separate statistically significant P values and false discovery rates for each identified transcription factor. In red, transcription factors with significant association to the differential proteomes. (**b**) The Western blot on top shows C-MYC expression in the indicated AsPC-1 cell lines. The bar graph indicates the band intensity from the Western blot above. (**c**) The flow cytometry density plots show CD44/EpCAM expression in the indicated cell lines. Percentages within the graphs show the percentage of CD44-EpCAM double positive AsPC-1 cells. The scatter plot represents the same data from five independent experiments. Statistical comparisons were performed by ANOVA and pairwise Tukey’s tests. (**d**) Bar graph representing means and standard deviations of the number of the indicated cell lines transduced with the lentivector expressing active C-MYC and blasticidin resistance, after two weeks of selection. Statistical comparisons were performed by one-way ANOVA. (**e**) NANOG expression assessed by flow cytometry in the indicated cells lines. A well-defined NANO-positive population is indicated with an arrow. (**f**) AMPK expression by Western blot in the indicated cell lines. *, **, ***, indicate significant (*p* < 0.05), very significant (*p* < 0.01) and highly significant (*p* < 0.001) differences, respectively. Original uncropped Western blots and densitometry readings are shown in Figure S11.

AsPC-1 cells with silenced AKT1 presented most of the features resembling CSC characteristics. To identify potential transcription factors that could regulate the adaptation to AKT1 silencing that could drive the CSC phenotype either by activation or inhibition, we used the TfactS algorithm with differentially regulated proteins as inputs. We used both the whole differential AsPC-1-shAKT1 proteome and also its differential mitochondrial proteome. In both cases, several transcription factors related to cell pluripotency were significantly predicted, but the top significant altered regulator in both instances was C-MYC (Figure 7a). It has been previously shown that C-MYC activities are downregulated in pancreatic cancer CSCs [4]. Hence, we first assessed C-MYC expression by Western blot in AsPC-1 cells with silenced AKT isoforms (Figure 7b). In agreement with a previous report in pancreatic CSCs [4], our results show lower C-MYC expression also in our adapted cells with silenced AKT isoforms. Hence, to find out if C-MYC functional inhibition was associated to acquisition of a CSC phenotype, constitutively active C-MYCT58A mutant [27] or the dominant negative C-MYCΔHLH mutant [28] were stably expressed in AsPC-1 cells. Expression of constitutively active C-MYC significantly down-modulated co-expression of CD44/EpCAM (Figure 7c) and also exhibited a significant decrease in mitochondrial mass as ascertained by MG staining (Figure 7c). Interestingly, expression of dominant negative C-MYC was not sufficient to drive AsPC-1 de-differentiation. To find out if C-MYC inhibition was a requirement for AsPC-1 cells adapted to AKT1 silencing, these cells were transduced with a lentivector expressing constitutively active C-MYC T58A together with blasticidin resistance, followed by blasticidin selection. While AsPC-1 control cells could be selected and grown with active C-MYC, it was not possible to select viable AsPC-1 cells adapted to AKT1 silencing with active C-MYC (Figure 7d). These results show that C-MYC inactivation was absolutely required for survival of pancreatic cancer cells adapted to AKT1 silencing. In agreement with previous studies on pancreatic CSCs [4], our results suggest that C-MYC activities keep AspC1 cells in a differentiated state, but we found that C-MYC inhibition was not sufficient to drive the CSC phenotype although it was necessary for viability of undifferentiated cells.

Hence, we evaluated the expression of transcription factors that had been previously associated to CSC differentiation, resistance to radio-chemotherapy and disease relapse in various cancers. From a selection of targets, we found that NANOG was specifically expressed in AsPC-1 cells that had adapted to AKT1 silencing (Figure 7e). NANOG has been shown to be responsible for self-renewal and maintenance of pluripotency by transcriptionally repressing genes that drive cell differentiation [29], and its expression is associated to resistance to conventional therapies [30]. Then, we wondered whether known factors regulating mitochondrial biogenesis and NANOG expression could be overexpressed in pancreatic cancer cells as adaptation to AKT signaling [31]. We found no evidence in our proteomic data on expression of proteins such as NRF-1, SIRTs or proteins related to AMPK signaling. However, AMPK/mTOR signaling regulates mitochondrial biogenesis, and there is evidence that AMPK regulates NANOG expression in embryonic stem cells [32,33]. As a result of adaptation, we observed mTOR upregulation (Figure 2f) and AMPK-alpha upregulation in cells that had adapted to silencing of AKT isoforms (Figure 7f).

### 2.6. In Vivo AKT Inhibition Together with Metformin Treatment Increases Therapeutic Efficacy

**Figure 8:**
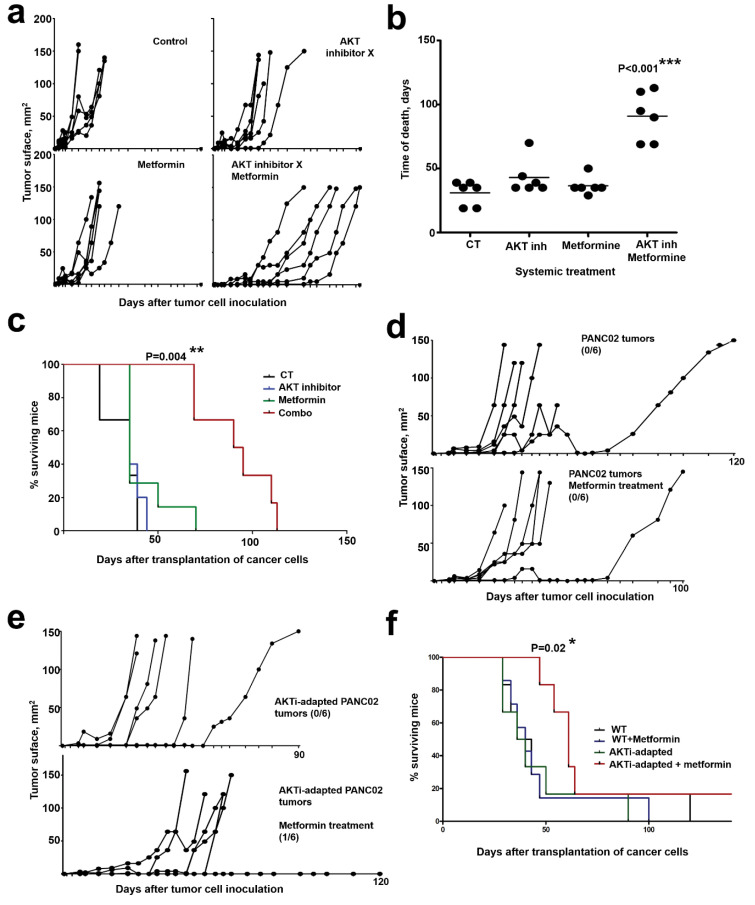
In vivo targeting of AKT and mitochondrial activities synergize in counteracting tumor growth. (**a**) PANC-02 tumor growth in individual mice (*n* = 6) from the indicated treatment groups. Drugs were administered twice subcutaneously. Control group, PBS-treated mice. (**b**) As in (**a**), but representing the time of death of each mice within the indicated treatment groups. Relevant statistical comparisons were performed with one-way ANOVA and Tukey’s a posteriori test, and shown within the graph. (**c**) Kaplan-Meier survival plot of the treatment groups, as indicated. (**d**) As in (**a**) but with metformin-only treatment in PANC02 tumors or with AKTi-adapted PANC02 tumors (**e**). The number of tumor-free mice/total mice are shown within the corresponding treatment groups. (**f**) Kaplan-Meier survival plot of the treatment groups as indicated. Relevant statistical comparisons were performed with. *, **, ***, indicate significant (*p* < 0.05), very significant (*p* < 0.01) and highly significant (*p* < 0.001) differences.

Our results suggest that dual inhibition of AKT and mitochondrial activities in vivo should inhibit tumor progression more effectively than monotherapies, and prolong survival in pre-clinical models of pancreatic cancer. To test this hypothesis, we first subcutaneously transplanted murine pancreatic adenocarcinoma PANC02 cells into C57BL/6 mice, as PANC02 cells harbors K-RAS mutations and exhibit a similar behavior to human pancreatic cancer cells [34]. When tumor growth was apparent, mice were subcutaneously administered with suboptimal doses of AKT inhibitor X (10-DEBC hydrochloride, 40 mg/1 kg) and metformin (40 mg/1 kg). These doses are well below the lowest published doses in similar experimental models (100 mg/kg, thrice per week for AKT inhibitor and 100 mg/kg daily for metformin) [35,36]. A group of mice was also treated with their combination (20 mg AKT inhibitor X + 20 mg metformin/1 kg), administered twice per week. Mice administered with PBS were used as controls. Only the combo treatment significantly delayed tumor growth (Figure 8a), increased lifespan (Figure 8b) and conferred survival advantage (Figure 8c). Treatments with either AKT inhibitor or metformin as monotherapies did not show significant therapeutic efficacies.

Then, we adapted PANC02 cells in vitro to prolonged inhibition of the AKT pathway by incubation in the presence of progressively increasing concentrations of AKT inhibitor X (AKTi), until reaching a toxic concentration to non-adapted cells. Similar to AsPC-1 cells adapted to prolonged AKT1 silencing, AKTi-adapted PANC02 exhibited noticeable phenotypic changes, recovered proliferation capacities comparable to non-adapted cells and showed a significant increase in mitochondrial mass as assessed by MG staining. Groups of mice were then subcutaneously injected with non-adapted (Figure 8d) or AKTi-adapted PANC02 cells (Figure 8e), and tumors were allowed to grow before starting a suboptimal metformin-only therapeutic regime. Only metformin-treated mice harboring AKTi-adapted PANC02 tumors showed significantly delayed tumor growth (Figure 8e), increased lifespan and survival advantage (Figure 8f). These results confirm that adaptation to AKT inhibition conferred increased in vivo sensitivity to metformin.

## 3. Discussion

Pancreatic adenocarcinoma shows very poor survival after diagnosis, being the fourth most frequent cause of cancer-related death. Its treatment is mostly limited to conventional chemotherapy, while targeted therapies exhibit poor clinical outcomes due to progression of resistant cancer cell variants. An increasing number of studies are indicating that the appearance of treatment-resistant cancer cells is dependent on metabolic plasticity and acquisition of CSC characteristics [4,37].

We studied the mechanisms of metabolic adaptation of human pancreatic cancer cells to inhibition of AKT, a master regulator of tumor progression and self-renewal of CSCs [38]. However, while most studies use small molecule inhibitors that simultaneously target the three AKT isoforms, we decided to assess the degree implication of each one in the process of molecular adaptation. Instead of genetic ablation of each AKT isoform, these were silenced with RNA interference so as not to completely eliminate their activity. We found a novel mechanism of CSC differentiation by adaptation to inhibition of the AKT pathway. We extensively characterized CSC differentiation through cell growth kinetics; quantitative proteomics; phenotype; and metabolic experiments including dependence of mitochondria by Seahorse analyses, silencing of mitochondrial proteins, sensitivity to antibiotics that show selective activities for CSC in vivo, ALDH expression and activities and spheroid formation capacities. The limitation of our current study is the absence of experiments of tumor growth in nude mice.

The major conclusion from the present study is that human pancreatic cancer cells undergo profound metabolic reprogramming that drives the acquisition of stem cell-like properties at different degrees after adaptation to silencing of AKT isoforms. It is worth noting that, even though all cells increased mitochondrial mass, silencing of each isoform caused distinct alterations, with only adaptation to AKT1 silencing causing acquisition of full CSC characteristics. Our data agree with previous reports in which C-MYC activities are down-modulated in pancreatic CSC [4], while Noh and colleagues showed enrichment of stem-cell phenotypes in human cervical cancer cells by NANOG upregulation [39]. Here, we also found AMPK upregulation that could explain mitochondrial biogenesis and NANOG expression, and that C-MYC inhibition was a requirement for CSC viability. Cells adapted to AKT2 silencing were the most differentiated and had the lowest capacities for spheroid growth. Interestingly, deletion of combinations of AKT isoforms favored the development of spontaneous hepatocellular carcinoma in murine models, by a process of favoring liver injury [40]. It could be tempting to speculate that AKT removal could enhance CSC differentiation also in hepatocellular carcinoma. Interestingly, systemic deficiency in AKT1 and AKT2 activities disrupt glucose homeostasis through reduce circulation of leptin. It might be possible that cancer cells adapt to glucose disruption by increasing mitochondrial mass, and triggering in this way CSC differentiation [41].

Adaptation to AKT silencing was found to be dependent on mitochondria as shown by silencing of mitochondrial targets Seahorse experiments and increased sensitivity to mitochondria-targeted agents, in agreement with other studies on CSCs [42,43]. We also observed upregulated ALDH activity, which is a CSC marker in different cancers including pancreatic [44,45,46,47], as well as increased autophagy especially for cancer cells adapted to AKT1 and AKT3 silencing [48].

Our results have significant consequences and demonstrate that cytotoxic treatments targeting the mTOR/AKT pathway drive de-differentiation towards CSCs after a period of adaptation, without the need of modulating other factors such as a specific tumor environment or acquisition of further mutations. This result strongly suggests that combination therapies targeting the AKT-mTOR signaling axis together with mitochondrial targets may show increased therapeutic capacities in vivo. Our preclinical in vivo data with PANC02 cells indicate this with a combo of metformin and AKT inhibitor, which significantly delayed tumor growth, increased lifespan and conferred survival advantage. Similar to our results with human pancreatic cancer cells, adaptation of murine PANC02 cells to toxic concentrations of AKT inhibitor led to recovery of proliferation, increased mitochondrial mass and enhanced sensitivity to metformin in vivo.

Collectively, our data and previous studies support the use of combination therapies that eliminate cancer cells, while inhibiting CSCs arising from adaptation to cytotoxic therapies by targeting their mitochondrial metabolism. CSC differentiation is possibly one of the main mechanisms for tumor escape in pancreatic cancer. According to the results of others and our own results, it would be advisable to test combination therapies targeting mitochondria and AKT, even together with immune checkpoint inhibition. This approach would utilize simultaneously three different anti-tumor mechanisms, and it may also improve the low efficacies of immunotherapies in pancreatic cancer.

## 4. Materials and Methods

### 4.1. Cell Lines

293T were grown following standard procedures. Human pancreatic adenocarcinoma AsPC-1 cells, pancreatic carcinoma NP18 cells and human primary pancreatic adenocarcinoma BxPC3 cells were acquired from American Type Culture Collection (ATCC). Murine pancreatic adenocarcinoma PANC02 cells were a kind gift from Dr. Ignacio Melero. Cells were grown in RPMI supplemented with 10% fetal bovine serum (FBS) and 1% penicillin/streptomycin. Cell growth and survival were monitored in real time using iCELLigence and xCELLigence real-time cell analysis (RTCA ACEA Biosciences) by seeding 20,000 cells and 5000 cells, respectively. Effective concentration 50 (EC_50_) to metformin and tigecycline for AsPC-1 cells was calculated by RTCA using increasing concentrations as described [49]. PANC02 cells were adapted to toxic concentrations of AKT inhibitor X (10-DEBC hydrochloride CAS number 925681-41-0. MilliporeSigma, St Louis, MO, USA), by continuous growth with AKTi through a step-by-step increase in concentration until reaching toxic concentrations (50 µM) to non-adapted cells during the course of one month.

Spheroid formation assays were performed as described [26]. Briefly, cells were trypsinized to obtain single cell suspension, counted, washed with PBS and resuspended in DMEM-F12 medium supplemented with FGF and B27 at density 2 × 10^3^ cells/mL. Cells were grown for 24 days in nonadherent plates (TC 6-well plate, suspension F. Sarstedt AG & Co, Nümbrecht, Germany). Afterwards, cells were collected, trypsinised, diluted and new cultures were set. These steps were repeated and cultures analyzed for spheroid numbers and sizes.

### 4.2. Lentivector Construction, Production and Cell Transduction

Coding sequences for short hairpin ARNs were designed using Clontech’s bioinformatic tool (http://bioinfo.clontech.com/rnaidesigner/). These sequences were the following: shAKT1: CGCGTGACCATGAACGAGTTTCTCGAGAAACTCGTTCATGGTCACGCGTTTTTG; shAKT2: CGGCTCCTTCATTGGGTACAACTCGAGTTGTACCCAATGAAGGAGCCGTTTTTG; shAKT3: GTAGTCCAACTTCACAAATTGCTCGAGCAATTTGTGAAGTTGGACTACTTTTTTG; shHARS2: ATTAACCCAGCTGCACTATTGTTCAAGAGACAATAGTGCAGCTGGGTTAATTTTTTTG; shSSBP1: GCATGGCACAGAATATCAGTATTTCAAGAGAATACTGATATTCTGTGCCATGTTTTTTG; and shTFB2M:GCCCAAAGCGTAGGGAATTATTTTCAAGAGAAATAATTCCCTACGCTTTGGGTTTTTTG. Coding sequences for shRNAs were cloned into the pSIREN lentivector platforms [50]. The sequence encoding the fusion protein mCherry-GFP-LC3B was synthetized (GeneArt^TM^ Thermo Fisher, Regensburg, Germany) and cloned into pDUAL-Puro and pDUAL-Blast lentivectors [50] under the transcriptional control of the SFFV promoter.

Lentivector production and titration were carried out as described elsewhere [50]. Transductions of cell lines were performed with a multiplicity of transduction of 10. Transduced cells were selected with appropriate concentrations of puromycin (Gibco^TM^ Thermo Fisher Scientific, Waltham, MA, USA) or blasticidin (Gibco^TM^ Thermo Fisher Scientific, Waltham, MA, USA). AsPC-1-mCherry-GFP-LC3B cells were further cloned by limiting dilution.

### 4.3. Mitochondrial Staining and Respiration

Mitochondria were stained with MitoTracker Green FM (Invitrogen^TM^ Thermo Fisher Scientific. Waltham, MA, USA) as recommended by the manufacturers. Briefly, cells were incubated at 37 °C with pre-warmed MitoTracker staining solution at a concentration of 10 nM for 30 min, washed with culture medium and fluorescence was quantified by optical microscopy using Cytation™ 5 (Biotek. Winooski, VT, USA). Mitochondrial respiration was evaluated using Agilent SeaHorse XF, as described with Agilent SeaHorse XF Cell Mito Stress [51]. ATP-linked respiration was measured in the presence of oligomycin. Maximum respiratory capacity was quantified by the addition of carbonyl cyanide-4 (trifluoromethoxy) phenylhydrazone (FCCP). Non-mitochondrial respiration was measured in the presence of rotenone/antimycin A.

### 4.4. Autophagy

AsPC1-mGLC3B modified with the indicated shRNAs were grown in 24-well plates for image captures. Representative images of living cells were captured with Cytation 5 fluorescence microscope (20×). Four fields by condition were taken for analyses using filters for mCherry and GFP fluorescence. Cytation 5 software was used to identify cellular events (10–100 µm) with GFP fluorescence as reference. Green and red mean fluorescences were quantified for these events and the ratio between mCherry and GFP fluorescence signals was calculated.

### 4.5. Immunoblot

Immunoblots were performed as previously described [50]. The following antibodies were used: anti-AKT1 (2H10), anti-AKT2 (L79B2), anti-AKT3 (L47B1), anti-mTOR (7C10) and anti-AMPK-alpha (2B7), purchased from Cell Signaling (Danvers, MA, USA); anti-HADHA, anti-VDAC1, anti-HARS2 and anti-SSBP1 obtained from Abcam (Cambridge, UK); anti-cMYC purchased from Invitrogen; anti-β-actin and anti-LC3B obtained from Sigma; and HRP anti-mouse and anti-rabbit antibodies purchased from Dako (Jena, Germany). Densitometry readings were performed with ImageJ (SYBYL Project, Newcastle, UK) by normalizing with background.

### 4.6. Flow Cytometry

Surface and intracellular staining were performed using routine protocols as described before [52]. The following antibodies were used: Ki67-APC (Biolegend, San Diego, CA, USA), CD44-APC and CD326/EpCAM-APCVio779 from Miltenyi Biotec (Bergisch Gladbach, Germany). Apoptosis was evaluated by Annexin V/Iodure Propidium staining using the Annexin V-FITC Apoptosis Detection Kit (Invitrogen™ eBioscience™).

ALDH activity was measured by flow cytometry with ALDEFLUOR (StemCell Technologies, Vancouver, BC, Canada) as recommended by the manufacturer. Briefly, 10 × 10^5^ cells were suspended in Aldefluor assay buffer containing an ALDH substrate (BODIPY-aminoacetaldehyde-diethyl acetate, BAAA-DA) and incubated at 37  °C for 45 min. A negative control for each sample was generated by incubation with the ALDH inhibitor diethylaminobenzaldehyde. Cells were washed with the Aldefluor buffer and analyzed by flow cytometry in a flow cytometer (BD Biosciences, San Jose, CA, USA).

### 4.7. Proteomics

A shotgun comparative proteomic analysis of cellular proteomes using iTRAQ (isobaric Tags for Relative and Absolute Quantitation) was performed following standard procedures [53]. Protein extracts were precipitated with methanol/choloroform and pellets dissolved in TEAB 0.5M and Urea 6M. Protein quantitation was performed with the Bradford assay kit (Bio-Rad, Hercules, CA, USA). iTRAQ labeling of each biological condition (in duplicates) was performed according to the manufacturer’s protocol (Sciex, Warrington, UK). Each tryptic digest was labeled with one isobaric amine-reactive tags as follows: Tag113, control cells A; Tag114, control cells B; Tag115, shAKT1-A; Tag116, shAKT1-B; Tag117, shAKT2-A; Tag118, shAKT2-B; Tag119, shAKT3-A; and Tag121, shAKT3-B. 

To increase proteome coverage, the peptide pool was fractionated by Pierce Strong Ion Exchange Spin Columns (Thermo Fisher, Waltham, MA, USA). Peptides were separated by reverse phase chromatography using an Eksigent nanoLC ultra 2D pump fitted with a 75 μm ID column (Eksigent 0.075 × 250). Eluting peptides from the column were analyzed using an Sciex 5600 Triple-TOF system. Data were acquired upon a survey scan performed in a mass range from 350 m/z up to 1250 m/z in a scan time of 250 ms. The top 25 peaks were selected for fragmentation. Minimum accumulation time for MS/MS was set to 75 ms giving a total cycle time of 2.1 s. Product ions were scanned in a mass range from 100 m/z to 1500 m/z and excluded for further fragmentation during 15 s. The mass spectrometry proteomics data have been deposited to the ProteomeXchange Consortium (http://proteomecentral.proteomexchange.org) via the PRIDE partner repository with the dataset identifier PXD020673.

### 4.8. Data Analysis

Data files were processed using ProteinPilot™ 5.0 software from Sciex (Warrington, UK) which uses the algorithm Paragon™ (v.4.0.0.0) [54] for database search and Pro Group™ Sciex (Warrington, UK) for data grouping and searched against Uniprot Human database (September 2016, 70550 entries). The search parameters allowed for cysteine modification by MMTS and biological modifications programmed in the algorithm. Reporter ion intensities were bias corrected for the overlapping isotope contributions from the iTRAQ tags. The peptide and protein selection criteria for relative quantitation were performed as previously described [55]. Several quantitative estimates provided for each protein by ProteinPilot were utilized: the fold change ratios of differential expression between labeled protein extracts and the *p*-value, representing the probability that the observed ratio is different than 1 by chance. A decoy database search strategy was used to estimate the false discovery rate (FDR) [56] and displayed results were those reporting a 1% Global FDR or better. Relative quantification and statistical analysis were provided by ProteinPilot software, with an additional 1.3-fold change cutoff for all iTRAQ ratios (ratio ≤0.77 or ≥1.3) to classify proteins as up- or downregulated. Proteins with iTRAQ ratios below the low range (0.77) were considered to be under-expressed, whereas those above the high range (1.3) were considered to be overexpressed. 

### 4.9. Bioinformatic Analyses

Identification of regulatory/metabolic networks was analyzed with STRING (Search Tool for the Retrieval of Interacting Genes) software (http://stringdb.org/) [57]. To infer differentially activated/deactivated pathways, proteomic data were further analyzed with QIAGEN’s Ingenuity^®^ Pathway Analysis (IPA) (QIAGEN Redwood City, CA, USA, www.qiagen.com/ingenuity).

### 4.10. In Vivo Experiments

C57BL/6 female mice were purchased from The Jackson Laboratory. Approval for animal studies was obtained from the Animal Ethics Committee of the University of Navarra (Pamplona, Navarra, Spain. Reference 077-19) and from the Government of Navarra. AKT inhibitor X (CAS 925681-41-0) and metformin hydrochloride were purchased from Sigma-Aldrich. AKT inhibitor X is an inhibitor of AKT phosphorylation, and presents an IC_50_ < 5 µM over the three AKT isoforms. Mice were inoculated with PANC02 cells and treated with suboptimal subcutaneous doses of AKT inhibitor X (1 mg/mouse twice a week), metformin (1 mg/mouse twice a week) or the combination (0.5 mg of each compound/mouse twice a week). PBS was used as vaccination vehicle for inhibitors, and PBS-treated mice were used as controls. Tumor size was measured twice a week. 

### 4.11. Statistics

Proteomic data were analyzed with Perseus after normalization, using ANOVA for multiple comparisons and t-test for pair-wise comparisons. All other variables under study were tested for normality by Kolmogorov-Smirnov test. Most of the variables were normally distributed with very low variability and homoscedasticity, so multi-comparisons were performed by one-way ANOVA followed by pairwise comparisons with Tukey’s tests when required. ALDH activity was compared by two-way ANOVA to eliminate inter-experimental variability. Survival was analyzed by Kaplan-Meier plots and log-rank tests. Time of death in murine experiments were compared by the non-parametric Kruskal-Wallis test.

## 5. Conclusions

Pancreatic cancer cells survive a prolonged inhibition of the AKT pathway by de-differentiation into CSCs after a period of adaptation, without the need of external factors. This process is dependent on increased mitochondrial functions, which in turn sensitizes these cells to combo treatments targeting AKT and mitochondria. 

## Supplementary Materials

The following are available online at https://www.mdpi.com/2072-6694/12/8/2181/s1, Figure S1: Western blot anti-AKT1 (56 KDa); Figure S2: Western blot anti-AKT2; Figure S3: Western blot anti-mTOR (protein size 290 KDa); Figure S4: Western blot anti-HARS2 (protein size 8 KDa); Figure S5: Western blot anti-TFB2M (protein size 45 KDa); Figure S6: Western blot anti-SSBP1; Figure S7: Western blot anti-VDAC1 (31 KDa); Figure S8: Western blot anti-HADHA (83 KDa); Figure S9: Western blot anti-ALDH1A1 (56 KDa); Figure S10: Western blot anti-ALDH2 (44 KDa); Figure S11: Western blot anti-c-MYC (57 KDa); Figure S12: Western blot anti-beta actin (About 40 KDa); Figure S13: Western blot anti-beta actin (About 15 KDa)
