# Supplementary material for: Profound Reprogramming towards Stemness in Pancreatic Cancer Cells as Adaptation to AKT Inhibition
Source: Cancers (Basel). 2020 Aug 5;12(8):2181. doi: 10.3390/cancers12082181 (PMC7464748; doi:10.3390/cancers12082181)
Supplement: Supplementary file 1 [file cancers-12-02181-s001.pdf]

Supplementary Materials

Profound reprogramming towards stemnes in pancreatic cancer cells as adaptation to AKT inhibition

Hugo Arasanz, Carlos Hernández, Ana Bocanegra, Luisa Chocarro, Miren Zuazo, Maria Gato, Karina Ausin, Enrique Santamaría, Joaquín Fernández-Irigoyen, Gonzalo Fernandez, Eva Santamaria, Carlos Rodríguez, Idoia Blanco-Luquin, Ruth Vera, David Escors and Grazyna Kochan

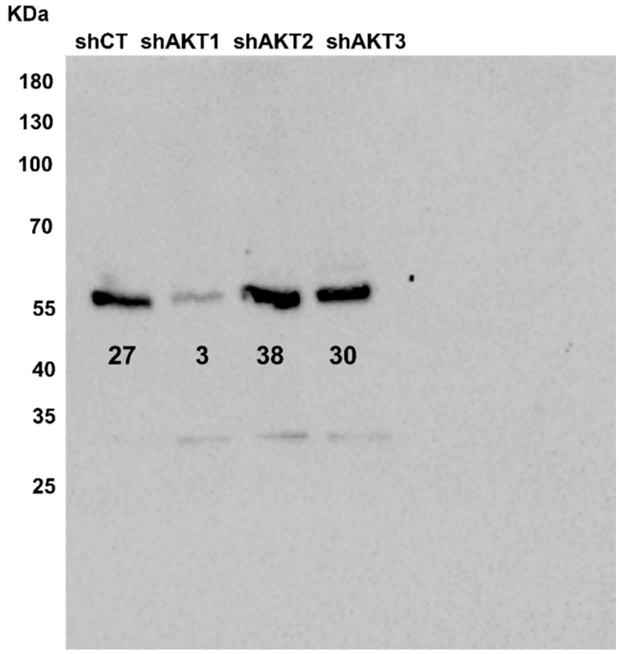

Figure S1. Western blot anti-AKT1 (56 KDa). The Western blot corresponds to the one shown in Figure 1a, developed with anti-AKT1. On the left, shown are the molecular weight markers. On the bottom, the normalized band densities corresponding to AKT1, as measured with ImageJ.

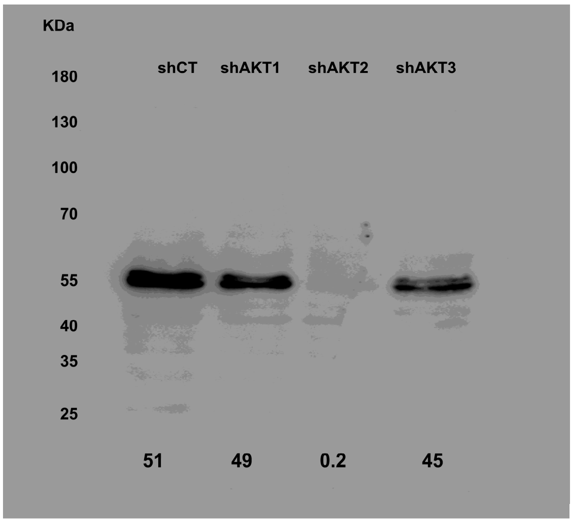

Figure S2. Western blot anti-AKT2. The Western blot corresponds to the one shown in Figure 1a, developed with anti-AKT2. On the left, shown are the molecular weight markers. On the bottom, the normalized band densities corresponding to AKT2, as measured with ImageJ.

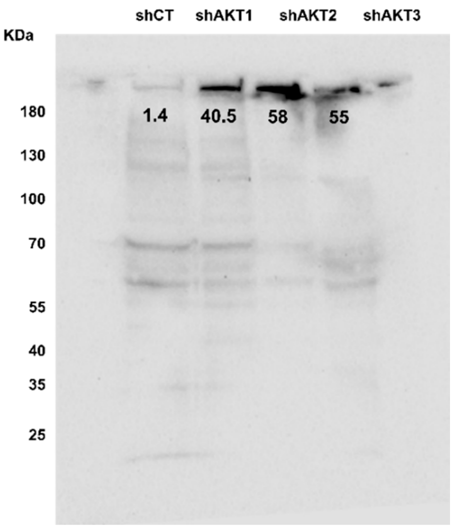

Figure S3. Western blot anti-mTOR (protein size 290 KDa). The Western blot corresponds to the one shown in Figure 2f, developed with anti-mTOR. On the left, shown are the molecular weight markers. On the bottom, the normalized band densities corresponding to mTOR, as measured with ImageJ.

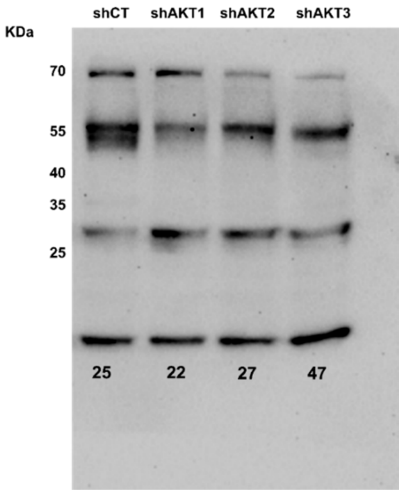

Figure S4. Western blot anti-HARS2 (protein size 8 KDa). The Western blot corresponds to the one shown in Figure 2f, developed with anti-HASR2. On the left, shown are the molecular weight markers. On the bottom, the normalized band densities corresponding to HARS2, as measured with ImageJ.

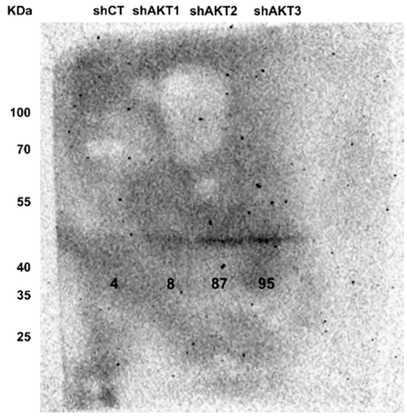

Figure S5. Western blot anti-TFB2M (protein size 45 KDa). The Western blot corresponds to the one shown in Figure 2f, developed with anti-TFB2M. On the left, shown are the molecular weight markers. On the bottom, the normalized band densities corresponding to TFB2M, as measured with ImageJ.

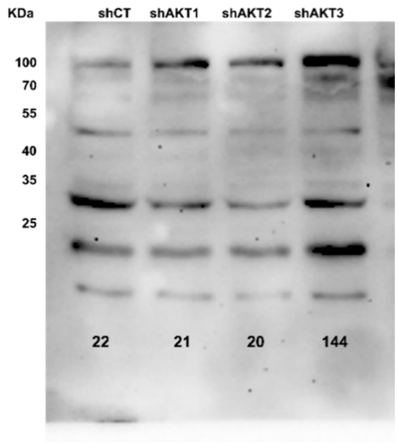

Figure S6. Western blot anti-SSBP1. The Western blot corresponds to the one shown in Figure 2f, developed with anti-SSBP1. On the left, shown are the molecular weight markers. On the bottom, the normalized band densities corresponding to SBBP1, as measured with ImageJ.

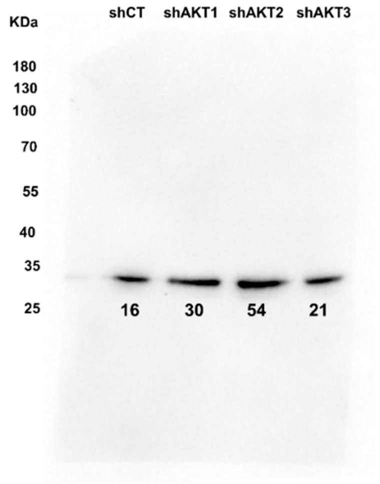

Figure S7. Western blot anti-VDAC1 (31 KDa). The Western blot corresponds to the one shown in Figure 2f, developed with anti-VDAC1. On the left, shown are the molecular weight markers. On the bottom, the normalized band densities corresponding to VDAC1, as measured with ImageJ.

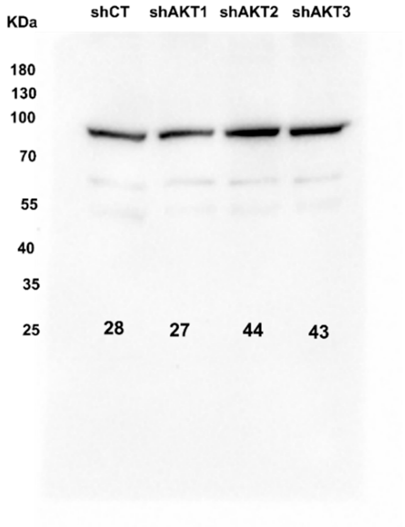

Figure S8. Western blot anti-HADHA (83 KDa). The Western blot corresponds to the one shown in Figure 2f, developed with anti-HADHA. On the left, shown are the molecular weight markers. On the bottom, the normalized band densities corresponding to HADHA, as measured with ImageJ.

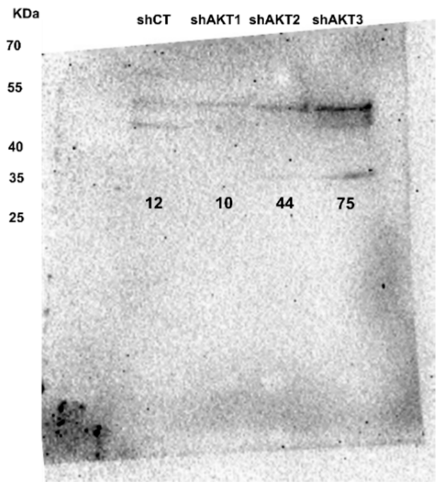

Figure S9. Western blot anti-ALDH1A1 (56 KDa). The Western blot corresponds to the one shown in Figure 5f, developed with anti-ALDH1A1. On the left, shown are the molecular weight markers. On the bottom, the normalized band densities corresponding to anti-ALDH1A1, as measured with ImageJ.

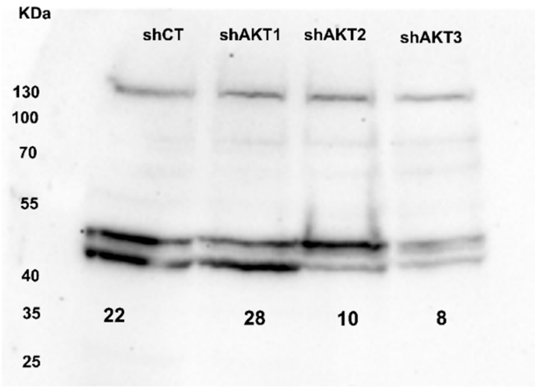

Figure S10. Western blot anti-ALDH2 (44 KDa). The Western blot corresponds to the one shown in Figure 5f, developed with anti-ALDH2. On the left, shown are the molecular weight markers. On the bottom, the normalized band densities corresponding to anti-ALDH2, as measured with ImageJ.

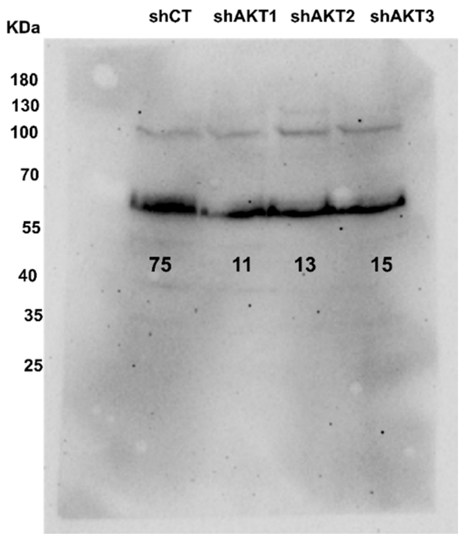

Figure S11. Western blot anti-c-MYC (57 KDa). The Western blot corresponds to the one shown in Figure 7, developed with anti-c-MYC. On the left, shown are the molecular weight markers. On the bottom, the normalized band densities corresponding to anti-c-MYC, as measured with ImageJ.

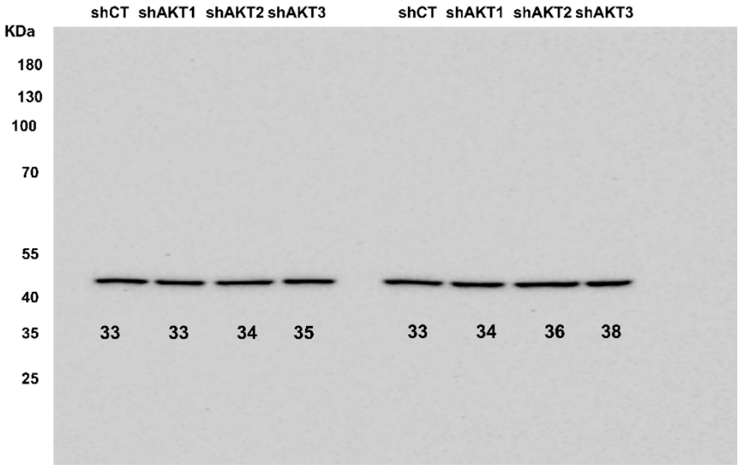

Figure S12. Western blot anti-beta actin (About 40 KDa). The Western blot corresponds to the one shown in Figure 6a, developed with anti-beta actin. On the left, shown are the molecular weight markers. On the bottom, the normalized band densities corresponding to anti-b-actin, as measured with ImageJ.

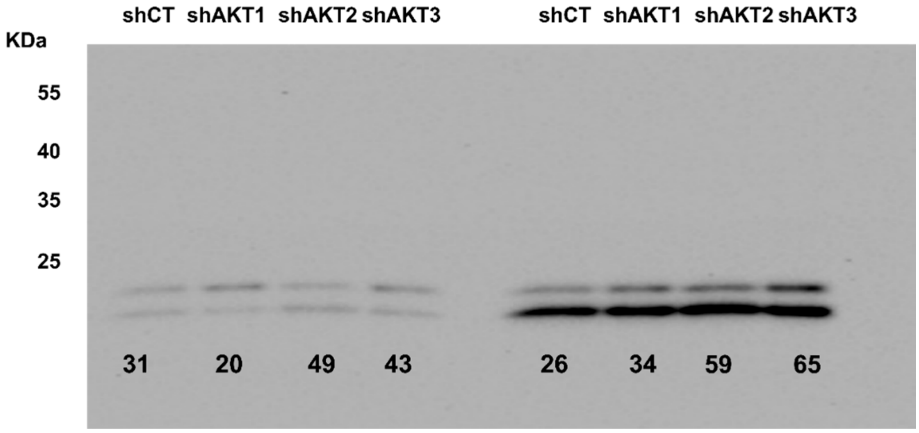

Figure S13. Western blot anti-beta actin (About 15 KDa). The Western blot corresponds to the one shown in Figure 6a, developed with anti-LC3B. On the left, shown are the molecular weight markers. On the bottom, the normalized band densities corresponding to anti-LC3B, as measured with ImageJ.

© 2020 by the authors. Licensee MDPI, Basel, Switzerland. This article is an open access article distributed under the terms and conditions of the Creative Commons Attribution (CC BY) license (http://creativecommons.org/licenses/by/4.0/).
